# Transcriptome sequencing and phylogenomic resolution within Spalacidae (Rodentia)

**DOI:** 10.1186/1471-2164-15-32

**Published:** 2014-01-17

**Authors:** Gong-Hua Lin, Kun Wang, Xiao-Gong Deng, Eviatar Nevo, Fang Zhao, Jian-Ping Su, Song-Chang Guo, Tong-Zuo Zhang, Huabin Zhao

**Affiliations:** 1 Key Laboratory of Adaptation and Evolution of Plateau Biota, Northwest Institute of Plateau Biology, Chinese Academy of Sciences, Xining 810008, China; 2 State Key Laboratory of Grassland Agro-ecosystem, College of Life Science, Lanzhou University, Lanzhou, China; 3 Graduate University of the Chinese Academy of Sciences, Beijing 100049, China; 4 Institute of Evolution, University of Haifa, Mount Carmel, Haifa 31905, Israel; 5 Department of Zoology, College of Life Sciences, Wuhan University, Wuhan 430072, China

**Keywords:** Spalacidae, Phylogenomics, Transcriptome, Mitochondrial genome, Subterranean rodents

## Abstract

**Background:**

Subterranean mammals have been of great interest for evolutionary biologists because of their highly specialized traits for the life underground. Owing to the convergence of morphological traits and the incongruence of molecular evidence, the phylogenetic relationships among three subfamilies Myospalacinae (zokors), Spalacinae (blind mole rats) and Rhizomyinae (bamboo rats) within the family Spalacidae remain unresolved. Here, we performed *de novo* transcriptome sequencing of four RNA-seq libraries prepared from brain and liver tissues of a plateau zokor (*Eospalax baileyi*) and a hoary bamboo rat (*Rhizomys pruinosus*), and analyzed the transcriptome sequences alongside a published transcriptome of the Middle East blind mole rat (*Spalax galili*). We characterize the transcriptome assemblies of the two spalacids, and recover the phylogeny of the three subfamilies using a phylogenomic approach.

**Results:**

Approximately 50.3 million clean reads from the zokor and 140.8 million clean reads from the bamboo ratwere generated by Illumina paired-end RNA-seq technology. All clean reads were assembled into 138,872 (the zokor) and 157,167 (the bamboo rat) unigenes, which were annotated by the public databases: the Swiss-prot, Trembl, NCBI non-redundant protein (NR), NCBI nucleotide sequence (NT), Gene Ontology (GO), Cluster of Orthologous Groups (COG), and Kyoto Encyclopedia of Genes and Genomes (KEGG). A total of 5,116 nuclear orthologous genes were identified in the three spalacids and mouse, which was used as an outgroup. Phylogenetic analysis revealed a sister group relationship between the zokor and the bamboo rat, which is supported by the majority of gene trees inferred from individual orthologous genes, suggesting subfamily Myospalacinae is more closely related to subfamily Rhizomyinae. The same topology was recovered from concatenated sequences of 5,116 nuclear genes, fourfold degenerate sites of the 5,116 nuclear genes and concatenated sequences of 13 protein coding mitochondrial genes.

**Conclusions:**

This is the first report of transcriptome sequencing in zokors and bamboo rats, representing a valuable resource for future studies of comparative genomics in subterranean mammals. Phylogenomic analysis provides a conclusive resolution of interrelationships of the three subfamilies within the family Spalacidae, and highlights the power of phylogenomic approach to dissect the evolutionary history of rapid radiations in the tree of life.

## Background

Subterranean mammals have received extensive attention for their specialized adaptations to life underground from scientists in different fields [1]. Three orders of mammals include subterranean species: the rodents, the insectivores, and the marsupials, which independently display adaptive convergence and divergence, are good subjects for comparative studies in morphology, ecology, physiology, ethology, and most recently, genetics [1,2].

All extant species of zokors (Myospalacinae, ~9 species), blind mole rats (Spalacinae, ~13 species), and bamboo rats (Rhizomyinae, ~17 species, including *Rhizomys*, *Cannomys* and *Tachyoryctes*) are typical subterranean mammals [3-6]. Because of their cryptic lifestyle and the morphological convergence associated with the subterranean life in tubular burrows [1], their systematic placement has been a long-standing puzzle [4]. Members of these subterranean rodents had been assigned to Muridae (mice and rats), Cricetidae (New World rats and mice, voles, hamsters, and relatives), and Rhizomyidae (bamboo rats and East African mole rats) [4]. Recently, overwhelming molecular evidence has suggested the cladistic reunion of zokors, bamboo rats and blind mole rats, and the monophyletic origin of the three groups that form the family Spalacidae has been generally accepted after a century of debate [4,7-13].

**Figure 1:**
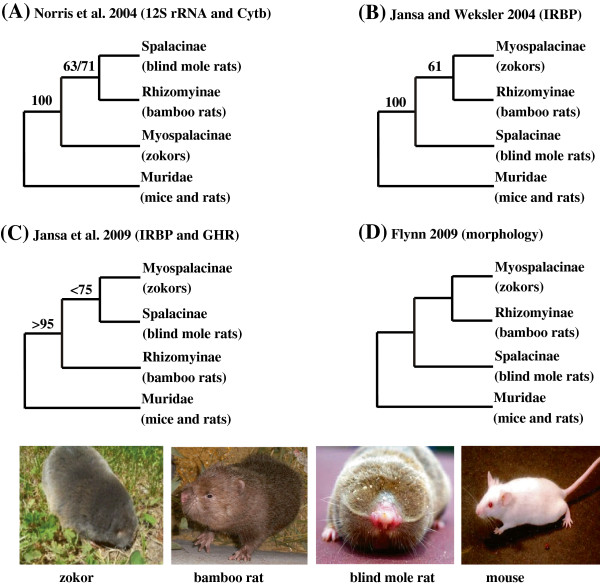
**Proposed phylogeny of three subfamilies Myospalacinae, Spalacinae and Rhizomyinae within the family Spalacidae.** Inferred from 12S rRNA and Cytb **(A)**, IRBP **(B)**, IRBP and GHR **(C)**, and morphology **(D)**. (Numbers above branches are bootstrap percentages. Note that there were no bootstrap values in the fossil study [8]).

Within the family Spalacidae, however, the phylogenetic affinities among the three subfamilies (Myospalacinae, Spalacinae, and Rhizomyinae) remain unclear [4]. Mitochondrial genes (*12S rRNA* and *Cytb*) recovered a sister clade consisting of Spalacinae and Rhizomyinae (Figure 1A) with moderate bootstrap support values of 63% or 71% depending on phylogenetic approaches, while Myospalacinae is basal relative to the two additional subfamilies with a strong support values of 100% [12]. By contrast, nuclear gene *IRBP* suggested that Myospalacinae is the sister to Rhizomyinae (Figure 1B), although the support (bootstrap percentage of 61%) is fairly weak [5]. By adding another nuclear gene, the concatenated genes of *IRBP* and *GHR* united Myospalacinae and Spalacinae to be a monophyletic group (Figure 1C) with a low bootstrap value, and Rhizomyinae is the sister to the monophyletic group [14]. Consistent with the phylogeny inferred from *IRBP* sequences (Figure 1C), paleontological evidence has shown that zokors share more morphological features with bamboo rats than with blind mole rats [8], supporting that Myospalacinae and Rhizomyinae are most closely related, while Spalacinae is more basal than the others (Figure 1D). These controversies prompt us to revisit the phylogeny of the three groups within the family Spalacidae using genomic data.

In this study, we employed the Illumina Hiseq 2000 platform and sequenced the transcriptomes of brain and liver of a plateau zokor (*Eospalax baileyi*) and a hoary bamboo rat (*Rhizomys pruinosus*). We compiled newly obtained transcriptome data from the zokor and the bamboo rat alongside the published transcriptome assembly from the Middle East mole rat [15], and provided a conclusive resolution of interrelationships of the three subfamilies within the family Spalacidae using a phylogenomic approach.

## Results

### Illumina sequencing and assembly

Total RNAs were isolated from brain and liver of an adult male plateau zokor. mRNA was purified by passing total RNA through a column of beads with oligo (dT), and was subsequently fragmented into short sequences of 200 bp. The fragmented mRNA was ligated with the adaptor mix and was transcribed to cDNA by reverse transcriptase before sequencing. The sequenced raw data were filtered to obtain clean reads by removal of adaptors, low-quality reads, and ambiguous reads. A total of 23,382,446 and 26,936,124 clean reads were obtained for the brain and liver of the zokor, respectively (GenBank BioProject number: PRJNA208780). As for the bamboo rat, 62,194,614 and 78,583,217 clean reads were obtained for the brain and liver, respectively (GenBank BioProject number: PRJNA211727) (Table 1). The reads of brain and liver of each species were pooled and were assembled into unigenes. Eventually, a total of 138,872 filtered transcripts (unigenes) of the zokor were obtained, and from which, 36,031 ORFs (Open Reading Frames) with a mean length of 975 bp were identified, while 157,167 unigenes were generated and 39,911 ORFs with a mean length of 1,102 bp were detected from the bamboo rat (Table 1). Details of the number of reads, mean length and the N50 values are given in Table 1.

**Table 1:** Details of the transcriptome sequences generated in this study (bp, base pair; ORF, Open Reading Frame; N50, N50 statistics)

	**Zokor**	**Bamboo rat**
**Clean reads**		
Brain	23,382,446	62,194,614
Liver	26,936,124	78,583,217
Total	50,318,570	140,777,831
**Unigenes**		
Number	138,872	157,167
Mean length (bp)	528	788
N50 (bp)	707	1,640
**Predicted ORFs**		
Number	36,031	39,911
Mean length (bp)	975	1,102
N50 (bp)	1,322	1,656

### Functional annotation and classification

In total, 50,550 (36.40%) and 55,581 (35.36%) unigenes of the zokor and the bamboo rat, respectively, were matched at least once by either of the public databases: Swiss-prot, Trembl, NCBI non-redundant protein (NR), NCBI nucleotide sequence (NT), Gene Ontology (GO), Cluster of Orthologous Groups (COG), and Kyoto Encyclopedia of Genes and Genomes (KEGG) (Table 2). For example, we used BLASTX to search all possible translations of unigenes of the zokor against the Swiss-prot protein database with an E-value of 1.0E-5, and obtained 31,077 hits, covering 22.38% of all unigenes (Table 2 and Additional file 1). Specifically, both GO (WEGO) and COG clustering showed similar distributions of the unigenes between the two species, suggesting the two transcriptomes are comparable (Additional files 2 and 3).

**Table 2:** Summary of unigene annotations of the two transcriptomes (Values in parentheses are percentages of all assembled unigenes in a given species)

**Database**	**Zokor**	**Bamboo rat**
Swiss-prot	31,077 (22.38%)	33,987 (21.62%)
Trembl	32,400 (23.33%)	35,299 (22.46%)
NR	33,890 (24.40%)	36,745 (23.38%)
NT	47,592 (34.27%)	52,129 (33.17%)
GO	27,726 (19.97%)	29,920 (19.04%)
COG	12,237 (8.81%)	12,273 (7.81%)
KEGG	10,390 (7.48%)	10,691 (6.80%)
All annotated	50,550 (36.40%)	55,581 (35.36%)

### Orthologous gene identification and multiple sequence alignment

The INPARANOID program was employed to identify orthologs based on two-way best genome-wide pairwise matches for each species pair [16]. The automatic clustering method implemented in the program can distinguish between orthologs and paralogs without using multiple alignments and phylogenetic trees [16,17]. All possible pairwise orthologs identified from the INPARANOID program were introduced into MULTIPARANID program to generate multiple-species orthologs [18]. All examined species include the plateau zokor, the hoary bamboo rat, the Middle East blind mole rat, and mouse which was used as an outgroup. Multiple sequence alignments of each ortholog were performed with the MACSE [19] and the PRANK [20] programs. Each alignment was check manually in MEGA 5 [21], alignments with poor quality or aligned region <100 bp were discarded. The resulting multiple sequence alignments include: (1) 5,116 individual alignments for each nuclear gene (Additional file 4), (2) 13 individual alignments for each protein-coding mitochondrial gene, (3) one alignment (5,541,534 bp in length) for the concatenated 5116 nuclear genes, (4) one alignment (715,762 bp in length, 12.92% of total length of the 5116 nuclear genes) for the concatenated fourfold degenerated sites (4D-sites) of the 5,116 nuclear genes, (5) one alignment (11,307 bp in length) for the concatenated 13 mitochondrial genes, and (6) one alignment (1,182 bp, 10.45% of total length of the 13 mitochondrial genes) for the concatenated 4D-sites of the 13 mitochondrial genes. Note that 4D-sites refer to the four-fold degenerate third codon positions of protein-coding genes at which any of the four nucleotide substitutions cannot result in an amino acid replacement, thus 4D-sites are a subset of synonymous sites that are supposed to be under no or very weak selection [22].

### Phylogenetic tree reconstruction

To avoid the confounding effects of sequence saturation in phylogenetic analysis, we assessed the substitution saturation with DAMBE5 [23]. Results showed that all the 500 randomly selected nuclear genes, the concatenated 4D-sites of the 5,116 nuclear genes and the concatenated 13 protein-coding mitochondrial genes obviously have experienced little substitution saturation (two tailed *P* value < 0.001). In contrast, the concatenated 4D-sites of 13 mitochondrial genes showed substantial substitution saturation (two tailed *P* value >0.05) and were excluded for further analysis.

We used both Maximum Likelihood (ML) and Bayesian Inference (BI) approaches for phylogenetic tree reconstruction. jModelTest2 program [24] was used to select the best-fitting substitution model for each alignment according to the Akaike information criterion and Bayesian information criterion [25]. For the 5,116 individual nuclear genes, totaling 70 models were selected as the best-fitting models for ML approach, the top five selected models were TrN + G, TrN + I, HKY + I, TIM3 + G, and TIM3 + I, covering 34.0% of the 5,116 cases. Totaling 13 models were chosen as the best-fitting models for Bayesian Inference (BI), the top five selected models were HKY + I, GTR + G, GTR + I, HKY + G, and HKY, accounting for 92.6% of the 5,116 cases (Additional file 5). For the 13 individual mitochondrial genes, the TIM2 models (TIM2 + I, TIM2 + G and TIM2 + I + G) were most common (8/13) for ML analysis, while the GTR models (GTR + I and GTR + G) were most popular (11/13) for BI approach (Additional file 5). In addition, the best-fitting models of the concatenated 4D-sites of the 5,116 nuclear genes were GTR + I + G for both ML and BI approaches.

**Figure 2:**
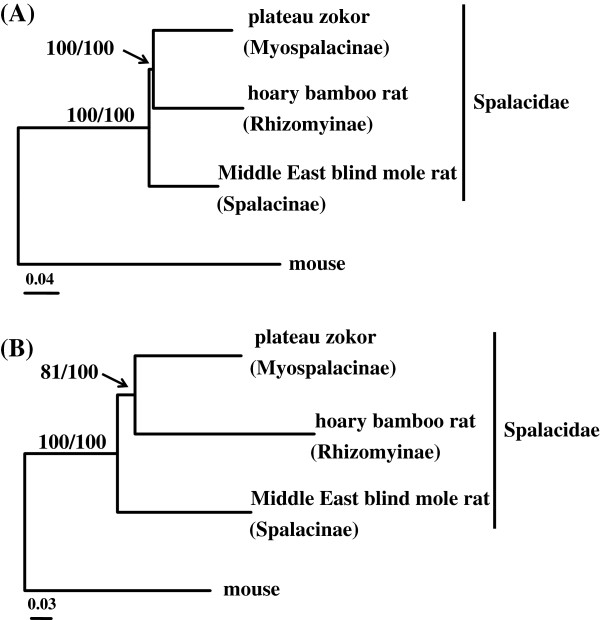
Phylogenetic relationships of the three subfamilies Myospalacinae, Spalacinae and Rhizomyinae inferred from the concatenated sequences of fourfold degenerate sites of the 5,116 nuclear genes (A) and the concatenated sequences of 13 protein coding mitochondrial genes (B).

Among the phylogenetic trees based on the 5,116 individual nuclear genes, 4,283 and 4,258 were supported with bootstrap values >50% for ML and BI methods, respectively. Moreover, the majority of phylogenetic trees derived from 13 individual mitochondrial genes were shown with bootstrap values >50% for both ML (12 of 13) and BI (11 of 13) approaches (Table 3). After counting the genes that support a tree with bootstrap values >50%, we found that ~39% of nuclear genes recovered a sister clade consisting of the zokor and the bamboo rat, and ~34% and ~27% of nuclear genes supported two other topologies (Table 2). Chi-square tests showed that the distributions of ML (χ^2^ = 92.84, *df* = 2, *P* <0.001) and Bayesian (χ^2^ = 93.39, *df* = 2, *P* <0.001) tree topologies were significantly deviated from random distribution, meaning that the majority of nuclear genes support a sister group of Myospalacinae and Rhizomyinae. By contrast, no significance was detected for either ML (χ^2^ =1.50, *df* = 2, *P* = 0.472) or Bayesian trees (χ^2^ = 1.27, *df* = 2, *P* = 0.529) inferred from 13 individual mitochondrial genes, possibly because of small number of genes used (Table 3). We also used a more stringent cutoff by counting the gene trees with boostrap values >70%. We found that 40% of nuclear genes supported a sister relationship between the zokor and the bamboo rat (Table 3), which is significantly deviated from random distribution (χ^2^ > 70, *df* = 2, *P* <0.001). In accordance with the individual gene analyses, the totality and the concatenated 4D-sites of the 5,116 nuclear genes revealed a same topology as the majority of nuclear genes and received 100% bootstrap support in both analyses (Figure 2A). Although the concatenated 13 protein-coding mitochondrial genes recovered the same phylogeny, the bootstrap values were relatively low (81% for ML tree and 100% for Bayesian tree) (Figure 2B). Strikingly, we observed a very short branch leading to Myospalacinae and Rhizomyinae (Figure 2), suggesting that the time between the divergence of Myospalacinae and Rhizomyinae and the first speciation before the divergence of Spalacidae is very short. The short period of time for the divergence of Spalacidae indicates a burst of rapid speciation occurred. This observation was previously revealed by a phylogenetic analysis inferred from the nuclear gene *IRBP*, although the monophyletic group comprising Myospalacinae and Rhizomyinae was weakly supported [5].

**Table 3:** Numbers of individual genes that support different phylogenetic tree topologies inferred from the Maximum Likelihood (ML) and Bayesian Inference (BI) approaches (only genes that support a tree with bootstrap values >50% and >70% were counted)

	**Tree topology**	**Nuclear genes**		**Mitochondrial genes**	
		**ML**	**BI**	**ML**	**BI**
Bootstrap values >50%	((Zokor, bamboo rat), blind mole rat)	1,676 (39.13%)	1,629 (38.26%)	6 (50.00%)	5 (45.46%)
	((Zokor, blind mole rat), bamboo rat)	1,445 (33.74%)	1,497 (35.15%)	3 (25.00%)	4 (36.36%)
	((Bamboo rat, blind mole rat), zokor)	1,162 (27.13%)	1,132 (26.59%)	3 (25.00%)	2 (18.18%)
	Total	4,283	4,258	12	11
Bootstrap values >70%	((Zokor, bamboo rat), blind mole rat)	835 (40.12%)	1,048 (39.28%)	5 (71.43%)	5 (50.00%)
	((Zokor, blind mole rat), bamboo rat)	732 (35.18%)	958 (35.91%)	2 (28.57%)	3 (30.00%)
	((Bamboo rat, blind mole rat), zokor)	514 (24.70%)	662 (24.81%)	0 (0.00%)	2 (20.00%)
	Total	2,081	2,668	7	10

## Discussion

When the genome sequence is unavailable, transcriptome sequencing is an effective way to obtain large numbers of transcripts. In this study, we present the first transcriptome data in zokors and bamboo rats using massively parallel mRNA sequencing. We perform Illumina sequencing of the brain which is the most complex organ in the body and the liver which plays a major role in metabolism. Although the raw data of the bamboo rat were much larger than that of the zokor, the results of sequence assembly, annotation, and classification of the two datasets were comparable. Furthermore, we provide a conclusive resolution of phylogenetic placement of the three subfamilies in the family Spalacidae. Phylogenetic analyses using various data coding schemes and analytical approaches overwhelmingly support that the subfamily Myospalacinae is more closely related to the subfamily Rhizomyinae than to the subfamily Spalacinae. Although we did not examine multiple individuals of each species, the genetic variations within species should not affect our phylogenetic resolution at subfamily levels.

Traditional sanger sequencing technology produces longer EST (expressed sequence tag) sequences with low throughput, which becomes little advantages when new assembly programs can handle large throughput short sequences [26,27]. Further, traditional transcriptome studies generally use a cDNA library of one tissue, while transcriptome sequencing based on NGS methods can work on a normalized cDNA library comprising multiple tissues and individuals, which would considerably reduce the sequencing cost. Hence, the cost- and time-effective NGS technologies will provide more comprehensive transcriptome information and facilitate the transcriptome studies, particularly in less-studied species. Although the large-scale transcriptome sequencing was used to sequence cDNA pools derived from *Spalax galili*[15], a member of the subfamily Spalacinae, the transcriptome surveys on the members of the other two subfamilies within the family Spalacidae are virtually lacking. Our work provides a valuable genomic resource and will stimulate the further analysis of these taxa with exceptional scientific interest. For example, subterranean rodents are believed to have greater hypoxia tolerance than their aboveground relatives [1]. Upon examining 247 candidate genes involving in adaptation to high-altitude hypoxia [28], we identified 195 (79%) and 207 (84%) genes in the zokor and bamboo rat transcriptomes, respectively. This finding will facilitate future comparative analysis of these functional genes.

Before the emergence of molecular identification approaches, one determines taxonomic status and phylogenetic relationships of animals mainly rely on morphological characteristics. However, the evolutionary change of morphological characters is extremely complicated (even for a short evolutionary time), the phylogenetic trees derived from morphological data frequently remain controversial [29]. In contrast, since the evolutionary change of DNA follows a traceable pattern, it is possible to use a mathematical model to formulate the change and compare DNA sequences among different organisms [29]. As a result, molecular phylogenetics is expected to clarify the tree of life that has been difficult to be resolved by the classical approaches. Taking zokors as an example, these animals have been allied to several different muroid subfamilies including Rhizomyinae, Spalacinae, Arvicolinae, and Cricetinae based on morphological characteristics [12], while overwhelming studies using molecular phylogenetic approaches concordantly placed zokors into Spalacidae [5,9,12,14]. It should be noticed that random noise will generally lead to poorly resolved phylogenetic trees, primarily because the number of nucleotide substitutions of a few genes is small. This may be the major reason why the previous molecular phylogeny studies showed inconsistence of topology relationships among the three subfamilies in Spalacidae [5,8,12-14].

In this study, we used a large number of genes including 5,116 nuclear genes and all 13 mitochondrial protein coding genes to examine the phylogenetic affinities of the three subfamilies within Spalacidae. Our analyses based on various data sets overwhelmingly support that the Spalacinae had a highest probability to be a basal clade relative to others within Spalacidae, while Rhizomyinae and Myospalacinae form a sister group. However, the most important challenges of phylogenomics involve different tree reconstruction methods and substitution saturation that can influence the validity of phylogenetic placements [30,31]. To overcome the challenges, we used both ML and BI approaches based on both nuclear and mitochondrial genes, and found no incongruence between the two tree reconstruction approaches (Figure 2). Further, we performed saturation tests and excluded the saturated 4D-site concatenation of mitochondrial genes in our analyses. Interestingly, our proposed phylogeny of the three subfamilies within Spalacidae is accordant with that inferred from massive morphological analyses on fossils of Spalacidae [8]. The known Early Miocene rhizomyines are closer to the stem zokor morphotype, suggesting that Myospalacinae is more closely related to Rhizomyinae than to Spalacinae. Although we didn’t examine African mole rats (*Tachyoryctes*) that were previously considered to be a separate subfamily in Spalacidae, multiple lines of molecular evidence consistently supported a sister relationship between *Rhizomys* and *Tachyoryctes*, suggesting that *Tachyoryctes* should be incorporated into the Rhizomyinae [3-6]. Together, our phylogenomic analyses unambiguously resolve the phylogeny of the three subfamilies comprising the family Spalacidae.

Geographically, members of the subfamily Spalacinae are found in East Europe, West Asia, Near East, and North Africa, members of Myospalacinae inhabit Siberia and Northern China, while the Rhizomyinae include two geographically distant relatives (bamboo rats and East African mole rats) that are from Southeastern Asia and East Africa [32]. Our molecular phylogenetic evidence suggests that the common ancestor of Spalacidae firstly split into Spalacinae and Rhizomyinae + Myospalacinae clades in the Northern Asia adjacent to the Middle East, and the latter clade subsequently split into Rhizomyinae and Myospalacinae in Mongolia and Northern China [8]. Interestingly, although Rhizomyinae and Myospalacinae are more phylogenetically related, Myospalacinae appears to be more biologically similar with Spalacinae than with Rhizomyinae for certain traits. For example, Myospalacinae and Spalacinae are totally blinded, while Rhizomyinae retain their sight [32]. The resolution of phylogenetic relationships among the three subfamilies will provide a framework for evolutionary studies on convergence and divergence of these animals.

## Conclusions

To summarize, this work is the first report of transcriptome sequencing in zokors and bamboo rats, representing a valuable resource for future studies of comparative genomics in subterranean mammals. Phylogenomic analysis provides a conclusive resolution of interrelationships of the three subfamilies within the family Spalacidae, and highlights the power of phylogenomic approach to dissect the evolutionary history of rapid radiations in the tree of life.

## Methods

### Ethics statement

All experimental protocols were approved by the Animal Care and Use Committee of Northwest Institute of Plateau Biology, Chinese Academy of Sciences.

### Taxon sampling and RNA sequencing

One adult male plateau zokor was caught by a live trapping arrow from Datong County (N 37°7.5′, E 101°48.7′), Qinghai Province, China. One male adult hoary bamboo rat was bought from a local farmer in Kunming (N 25°2.2′, E 102°42.5′), Yunnan Province, China. The animals were humanely sacrificed to collect the brain and liver tissues. The fresh tissues were frozen in liquid nitrogen immediately after collection, and stored at −80°C refrigerator before use. Total RNA was isolated from the brain and liver using Trizol (Invitrogen, CA, USA) following the manufacturer’s protocols.

Illumina sequencing was performed commercially following manufacturer’s instructions. Briefly, magnetic beads with oligo(dT) were used to purify poly(A) mRNA from the total RNA. Subsequently, the mRNA was fragmented into small pieces (200–500 bp) at 94°C for exactly 5 minutes. The cleaved RNA fragments were reverse transcribed into first-strand cDNA using SuperScript II reverse transcriptase and random primers, the second-strand cDNA was generated with GEX second strand buffer, DNA polymerase, RNase H and dNTPs. These cDNA fragments were further proceeded with end repair and 3’ adenylated. Paired-end adapters were ligated to the 3′ adenylated cDNA fragments. cDNA fragments of ~200 bp were selected and enriched by 15 cycles of PCR amplification with Phusion DNA Polymerase. Finally, four cDNA libraries were constructed and sequenced bi-directionally (100 bp each direction) on an Illumina Genome Analyzer (HighSeq2000, Illumina, San Diego, CA).

### De novo assembly and unigene annotation

The reads of brain and liver of each species were merged and after removal of the low quality reads, the clean reads were assembled with the Trinity program [26]. The generated unigenes were further filtered and clustered using CD-HIT-EST program [33]. The filtered unigenes were then used for BLAST search and annotation against the NCBI NR/NT database, the Swiss-prot/Trembl database using an E-value cut-off of 1E-5. The XML files generated from NR blast results were loaded into the Blast2GO software for GO (gene ontology) annotation. The ORFs were extracted by the Perl script transcripts_to_best_scoring_ORFs.pl (TransDecoder) in the Trinity program package. The ORFs were then submitted to the KAAS (KEGG Automatic Annotation Server, http://www.genome.jp/tools/kaas/) for KEGG pathways annotation.

### Sequence processing

The unigenes of the blind mole rat unigenes were downloaded from GenBank under the accession number provided by [15]. The predicted peptide sequences (ORFs) of this species were extracted by the Perl script Transdecoder in the Trinity program package. The mouse (used as outgroup in the tree reconstruction) cDNA and protein sequences were downloaded from Ensembl (http://www.ensembl.org). Because of the differences of genetic coding between mitochondrial DNA and nucleotide DNA, the mitochondrial DNA in the transcriptomes will be excluded by ORF finding and will not confuse the mitochondrial DNA extracted directly from the mitochondrial genome. The complete mitochondrial genomes of these four species were downloaded from GenBank with accession numbers JN540033.1 (plateau zokor, *E. baileyi*), AJ416891.1 (blind mole rat, *S. ehrenbergi*), KC789518 (hoary bamboo rat, *R. pruinosus*), and AY172335.1 (mouse, *Mus musculus*). The 13 protein coding sequences of mitochondrial genomes were extracted according to the definitions in the GenBank files. Additionally, fourfold degenerate sites (4D-sites) were identified in codeml from PAML [34] as all differences at the third sites of a codon are synonymous. The 4D-sites of the 5,116 nuclear genes and 13 mitochondrial genes were then extracted and concatenated, respectively.

### Orthologous gene identification and sequence alignment

The pairwise orthologous peptides between each two species were identified by the INPARANOID program [16]. The pairwise orthologous genes of all the four species were introduced into the MULTIPARANOID program [18] to generate multiple-species orthologs. The orthologs not covering all the four species were discarded in further analyses. The corresponding cDNA sequences of these orthologs were extracted by cdbfasta program [35] with the sequence names and were written into a single fasta file for each orthologous gene. Each fasta file contains four orthologs of a target gene. The sequences were aligned using the MACSE [19] and PRANK [20] programs. A simple in house Perl script was used to work on batch jobs for each of the files. The 13 protein coding mitochondrial genes were aligned directly using ClustalW program implemented in the MEGA software. In order to exclude low quality alignments, the orthologous genes with the aligned region <100 bp were discarded for further analyses.

### Phylogenomic analysis

To examine the substitution saturation, we randomly selected 500 nuclear genes, and tested using DAMBE [23]. All of the 13 mitochondrial genes were tested manually using DAMBE. Moreover, the 4D-sites concatenation of nucleotide and mitochondrial genes were also tested for saturation. Furthermore, we used both Maximum Likelihood (ML) and Bayesian Inference (BI) approaches for phylogenetic tree reconstruction. jModelTest2 program [24] was used to select the best-fitting substitution model for each alignment according to the Akaike information criterion and Bayesian information criterion [25].

The PhyML version 3.1 [36] was used to reconstruct ML trees for each gene with bootstrap replicates of 100 which is generally considered as a reasonable number of replicates. Again, a simple in-house Perl script was used to execute batch jobs. For the concatenated 5116 nuclear genes and the concatenated 13 mitochondrial genes, the RAxML version 7.8.6 [37] that allows multiple partitions with a same model (partitioned by the different genes with the recommended GTR + G model) was used for ML tree reconstruction. The MrBayes version 3.1.2 [38] was used to reconstruct trees for each gene, and for the concatenated 5116 nuclear genes and the concatenated 13 mitochondrial genes (partitioned according to different models) with 500,000 generations, which are sufficient to meet the 0.01 criteria of standard deviation of split frequencies. A batch mode defined by the program itself (set autoclose = yes nowarn = yes) was used for tree reconstruction. Finally, Chi-square test in SPSS 20.0 program was used to see whether the distribution of tree topologies significantly deviate the random distribution.

### Availability of supporting data

The sequencing data has been deposited to the GenBank under BioProject numbers of PRJNA208780 and PRJNA211727. The phylogenetic data is available from the Dryad Digital Repository: http://doi.org/10.5061/dryad.gs135.

## Abbreviations

NR: NCBI non-redundant proteins; NT: NCBI nucleotide sequences; GO: Gene Ontology; COG: Cluster of Orthologous Groups; KEGG: Kyoto Encyclopedia of Genes and Genomes; NGS: Next-generation sequencing; 4D-sites: Fourfold degenerated sites; ML: Maximum Likelihood; BI: Bayesian Inference; EST: Expressed sequence tag.

## Competing interests

The authors declare that they have no competing interests.

## Authors’ contributions

GHL, TZZ, and HZ conceived and designed the experimental plan. XGD and FZ performed experiments. GHL, KW, JPS and HZ analyzed and interpreted the sequencing data. GHL, EN, TZZ and HZ wrote the paper. All authors read and approved the final manuscript.

## Supplementary Material

Additional file 1 **Swiss-prot annotations of the zokor and the bamboo rat transcriptomes, including unigene name, length, database sequence length, unigene alignment, database sequence alignment, annotation, E-value and identity.** Click here for file

Additional file 2 Functional classification of the unigenes of the zokor and the bamboo rat based on three main GO (Gene Ontology) categories: biology process, molecular function and cellular component. Click here for file

Additional file 3 COG (Cluster of Orthologous Groups) functional classification of the transcriptomes of the zokor and the bamboo rat. Click here for file

Additional file 4 Phylogenomic data set of 5,116 nuclear genes with aligned length of 5,541,534 bp. Click here for file

Additional file 5 Phylogenetic results of each of the 5,116 nuclear genes and 13 mitochondrial genes, including selected best models, consensus trees and bootstrap values. Click here for file
