# Supplementary figures and images for: Transcriptome sequencing and phylogenomic resolution within Spalacidae (Rodentia)
Source: BMC Genomics. 2014 Jan 17;15:32. doi: 10.1186/1471-2164-15-32 (PMC3898070; doi:10.1186/1471-2164-15-32)

# WEGO output

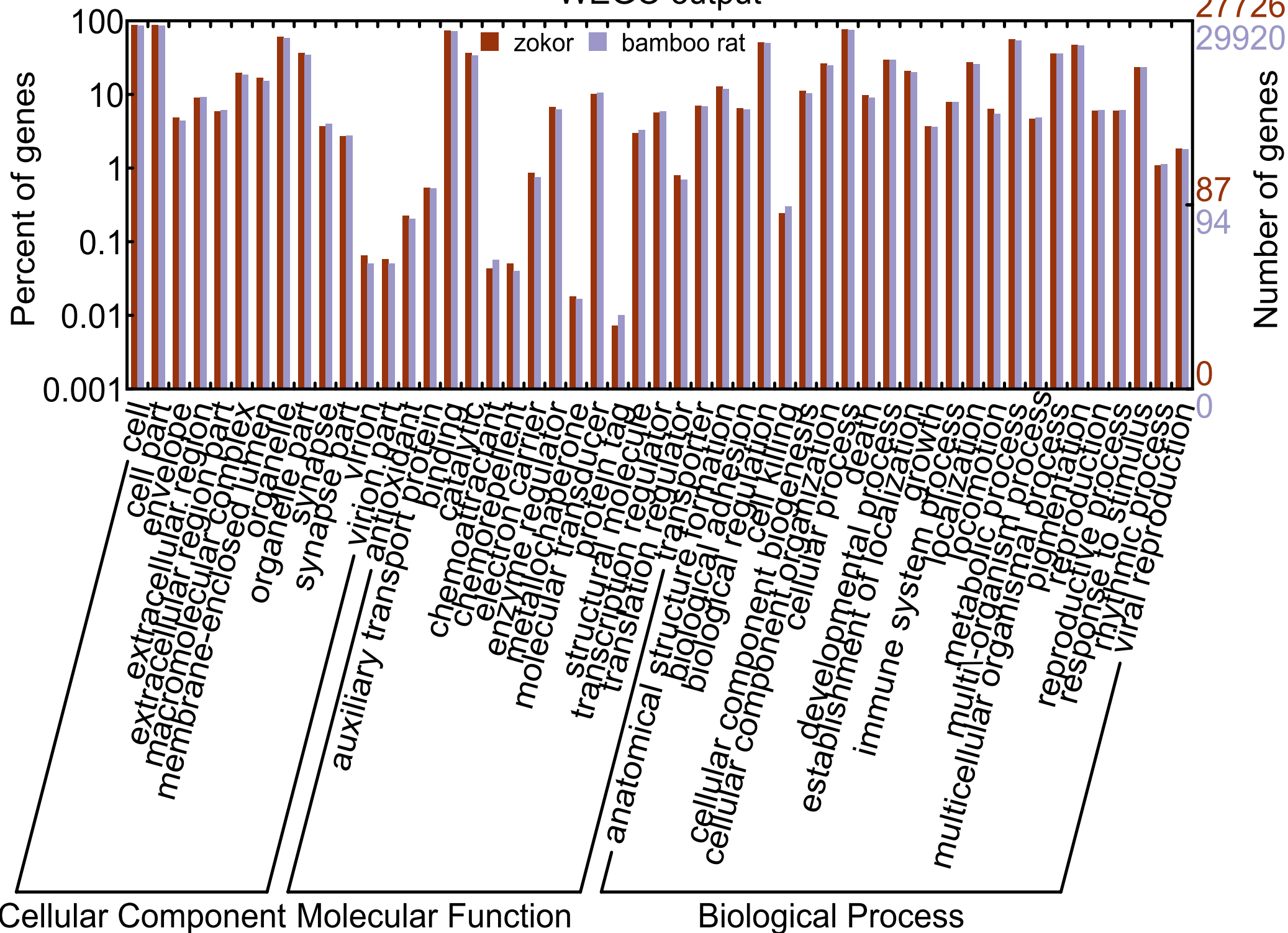

Supplement: Additional file 2 — Functional classification of the unigenes of the zokor and the bamboo rat based on three main GO (Gene Ontology) categories: biology process, molecular function and cellular component. [file 1471-2164-15-32-S2.pdf]
