# Supplementary material for: Transcriptome sequencing and phylogenomic resolution within Spalacidae (Rodentia)
Source: BMC Genomics. 2014 Jan 17;15:32. doi: 10.1186/1471-2164-15-32 (PMC3898070; doi:10.1186/1471-2164-15-32)
Supplement: Additional file 3 — COG (Cluster of Orthologous Groups) functional classification of the transcriptomes of the zokor and the bamboo rat. [file 1471-2164-15-32-S3.pdf]

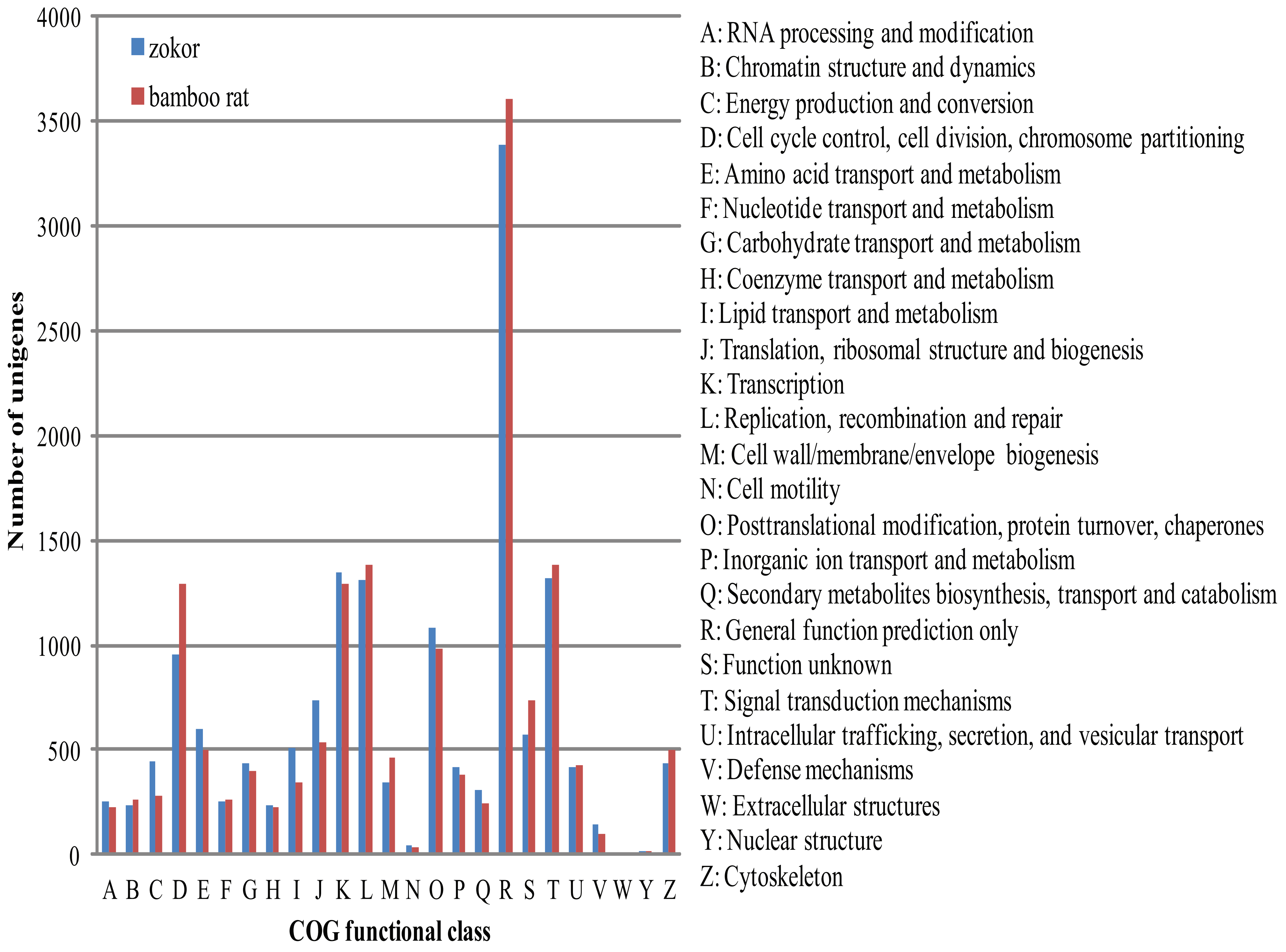
4000
3500
3000
2500
2000
1500
1000
500
0
Number of unigenes
zokor
bamboo rat
A B C D E F G H I J K L M N O P Q R S T U V W Y Z
COG functional class
A: RNA processing and modification
B: Chromatin structure and dynamics
C: Energy production and conversion
D: Cell cycle control, cell division, chromosome partitioning
E: Amino acid transport and metabolism
F: Nucleotide transport and metabolism
G: Carbohydrate transport and metabolism
H: Coenzyme transport and metabolism
I: Lipid transport and metabolism
J: Translation, ribosomal structure and biogenesis
K: Transcription
L: Replication, recombination and repair
M: Cell wall/membrane/envelope biogenesis
N: Cell motility
O: Posttranslational modification, protein turnover, chaperones
P: Inorganic ion transport and metabolism
Q: Secondary metabolites biosynthesis, transport and catabolism
R: General function prediction only
S: Function unknown
T: Signal transduction mechanisms
U: Intracellular trafficking, secretion, and vesicular transport
V: Defense mechanisms
W: Extracellular structures
Y: Nuclear structure
Z: Cytoskeleton
